# Notes and News

**Published:** 1899-07-15

**Authors:** 


### NOTES AND NEWS.

(Collected and Communicated.)

Several of the London hospitals will be closed during August. Amongst them are King's College Hospital and University Hospital.

Dr. Brown, Senior Honorary Consulting Physician to the Royal Preston Infirmary, has offered to defray the cost of an operating theatre for that institution. The expenditure is estimated at about £2,000.

The Cancer Society has commissioned Mr. Arthur C. Duffey, M.B., to proceed to America to report more especially as to the working of the State Laboratory for the study of cancer at Buffalo, N.Y.

The research expedition to West Africa, which is being sent out by the Liverpool School of Tropical Medicine, will probably start near the end of this month or the beginning of August.

The last remaining unused ward in the Great Northern Central Hospital has now been opened, raising the number of beds in the hospital to 160. This action is the direct result of the conditions appended to a grant from the Prince of Wales's Hospital Fund.

If anyone wishes to read a really graphic and picturesque account of cancer and all its works, let him take up the *Contemporary* for this month and read the article by Dr. Woods Hutchinson. As a piece of descriptive writing it is very well done, and we congratulate him upon it.

Big Ben has been made to hold his peace at night in consequence of the serious illness of the wife of the Sergeant-at-Arms, whose residence adjoins the clock tower. This is most proper. We have every sympathy with any sick person who has to lie within range of so powerful a bell during the still hours, and our sympathy is sufficiently wide to make it embrace many, besides Lady Erskine, who are deprived of nature's sweet restorer by the terrible and useless noises of the night, which, notwithstanding our pretended civilisation, we allow to disturb the hard-earned rest of thousands, and harass the dying beds of hundreds in this great city.

The foundation-stone for a new cottage hospital and nursing institute at Hinckley has been laid by Sir John F. L. Rolleston.

Another case of total extirpation of the stomach for carcinoma of the pylorus is reported, this time from Switzerland. The operation was performed by Dr. Kocher a month ago. The patient died three and a half days after the operation.

Arrangements have been made, says the New York *Medical Record*, for Dr. Doty, health officer of the port of New York, to use the yellow fever serum in Vera Cruz, where there is now an epidemic of yellow fever. The disease is prevalent also at Panama.

Among the matters to be discussed at the Italian Society of Laryngology, Otology, and Rhinology which will be held in Rome next October will be the question of the designation which ought to be applied to a specialist in oto-rhino-laryngology.

It is believed that in the forthcoming report of the Board appointed by the United States War Department to study the distribution of typhoid fever in army camps it will be shown that the disease was distributed by dust and by flies more than by water, which, it is believed, had but little to do with it.

The plague at Alexandria continues, although it has not, so far, affected a large number of people. What is important, however, is that a far larger proportion of those attacked have been Europeans than has been the case in India. When Europeans approximate to the natives in their health surroundings, as is the case with many of the Greeks, &c., in Alexandria, mere race is no protection.

At the annual meeting of the State Children's Aid Association, Dr. Barnardo drew attention to the high rate of mortality resulting from the aggregation of children under five years of age in large institutions. At his institution he had found that, notwithstanding every care, the mortality among the children when living together was 9 per cent.; but under the boarding-out system which he had adopted during the past 19 months, there had not been a single death among the 380 young children whom he had placed under the care of foster-parents. The cost per child boarded out was £13 1s. per annum.

The visit of the Duke and Duchess of York to Newton Abbot draws public attention to a part of the world which, well supplied as it may be by nature with all that makes for health and longevity, cannot be said to have been treated nearly so well by man. At the last meeting of the Newton Abbot Rural District Council, attention was drawn to the sanitary condition of Hennock, a place within the district; and, as indicating the condition in which the people live there, it was stated that there were only three closets for the use of 93 persons. This may not be a very important matter in a country place, but at least it stamps the district, and suggests that the amount of attention which is likely to be paid to other sanitary matters is not overwhelming.

